# The Therapeutic Potential of *Dalbergia pinnata* (Lour.) Prain Essential Oil in Alzheimer’s Disease: EEG Signal Analysis In Vivo, SH-SY5Y Cell Model In Vitro, and Network Pharmacology

**DOI:** 10.3390/biology13070544

**Published:** 2024-07-18

**Authors:** Sheng Qin, Jiayi Fang, Xin He, Genfa Yu, Fengping Yi, Guangyong Zhu

**Affiliations:** Department of Perfume and Aroma Technology, Shanghai Institute of Technology, Shanghai 201418, China; qinsheng0318@163.com (S.Q.); fnt18470537016@163.com (J.F.); 18018580892@163.com (X.H.); shyugenfa@163.com (G.Y.)

**Keywords:** *Dalbergia pinnata*, Alzheimer’s disease, essential oil, electroencephalography, SH-SY5Y cell, network pharmacology

## Abstract

**Simple Summary:**

Simple Summary: Plant essential oils are currently gaining increasing attention for their roles in mood regulation and neuroprotection. *Dalbergia pinnata* (Lour.) Prain (DP) is a traditional aromatic medicinal plant in China, primarily containing elemicin and methyl eugenol. Despite limited research, the potential neurological effects of aromatherapy are acknowledged, particularly in Alzheimer’s Disease. The pathogenesis of AD involves amyloid-beta (Aβ) deposition and Tau protein hyperphosphorylation, leading to neuronal dysfunction and inflammation. This study aims to document changes in brainwave power in male and female subjects following inhalation of DP essential oil (DPEO) and to investigate its impact on mood and brain function across genders. Additionally, the study examines the efficacy of DPEO in mitigating Aβ_1–42_-induced neurotoxicity using an in vitro Alzheimer’s Disease neural cell model.

**Abstract:**

Alzheimer’s disease (AD) is a neurodegenerative disorder that is projected by the WHO to affect over 100 million people by 2050. Clinically, AD patients undergoing long-term antipsychotic treatment often experience severe anxiety or depression in later stages. Furthermore, early-stage AD manifests with weakened α waves in the brain, progressing to diminished α and β waves in late-stage disease, reflecting changes in emotional states and disease progression. In this study, EEG signal analysis revealed that inhalation of *Dalbergia pinnata* (Lour.) Prain essential oil (DPEO) enhanced δ, θ, α and β wave powers in the frontal and parietal lobes, with a rising trend in the β/α ratio in the temporal lobe. These findings suggest an alleviation of anxiety and an enhancement of cognitive functions. Treatment of the AD SH-SY5Y (human neuroblastoma cells) cell model with DPEO resulted in decreased intracellular levels of Aβ, GSK-3β, P-Tau, IL-1β, TNF-α, IL-6, COX-2, OFR, and HFR, alongside reduced AchE and BchE activities and increased SOD activity. Network pharmacology analysis indicated a potential pharmacological mechanism involving the JAK-STAT pathway. Our study provides evidence supporting DPEO’s role in modulating anxiety and slowing AD pathological progression.

## 1. Introduction

*Dalbergia pinnata.* (Lour.) Prain (DP) is a plant native to tropical and subtropical regions. Local communities have traditionally used it to prevent respiratory, digestive, and cerebrovascular diseases. In Chinese traditional medicine, it has been employed for treating various traumatic injuries, such as burns and wounds [1], showcasing its robust cellular repair capabilities. Furthermore, recent studies have revealed that *Dalbergia pinnata* essential oil (DPEO) possesses potent antibacterial, antioxidant, tyrosinase inhibition, and anti-melanogenic activities [2]. However, there is limited reporting on its neuropharmacological effects.

Alzheimer’s disease (AD) is a common neurodegenerative cognitive disorder in the elderly. According to WHO predictions, by 2050, the global number of individuals affected by AD will exceed 100 million [3]. Modern epidemiological investigations suggest that the pathological characteristics of AD are highly associated with the deposition of amyloid-beta (Aβ) in the cerebral cortex and the excessive phosphorylation of Tau protein, leading to the formation of neurofibrillary tangles (NFT) [4]. These pathological products can result in multiple impairments in cerebral cortical functions (such as memory, thinking, orientation, comprehension, calculation, learning, and language) and cognitive deficits (aphasia and amnesia) [5,6].

APP (amyloid precursor protein) is expressed in neurons and glial cells, mediating cell adhesion, neuronal signaling, and neurotransmitter regulation [7]. It can be cleaved by α/β/γ-secretases into peptides ranging from 37 to 49 amino acids, which is a process known as amyloidogenic or non-amyloidogenic [8]. APP is cleaved by α-secretase via the non-amyloidogenic pathway, followed by γ-secretase cleavage. The resulting APPα fragment is soluble, non-toxic, and essential, with neuroprotective functions [8]. Conversely, β-secretase cleaves the C-terminal fragment of APP, generating neurotoxic Aβ peptides. Aβ_1–42_, with its highly hydrophobic C-terminus, triggers the aggregation of Aβ into insoluble structures, forming plaques that spread throughout the brain [9]. These plaques disrupt intercellular communication, leading to microglial activation, inflammation, neuronal death, and tissue damage in the brain [10].

The Tau protein is expressed in neurons, astrocytes, and oligodendrocytes. Under normal conditions, the Tau protein binds to microtubules as a component of the cellular cytoskeleton to stabilize synaptic functions [11]. However, abnormal hyperphosphorylation of Tau protein leads to microtubule structural abnormalities and the formation of neurofibrillary tangles (NFTs), resulting in intracellular aggregation [12]. Excessive phosphorylation of Tau protein and NFTs are associated with the pathogenesis of AD [13,14]. Furthermore, Aβ deposition can exacerbate the AD pathogenic mechanisms mediated by the Tau protein, thereby exacerbating NFTs, cognitive impairment, and dementia [15]. Additionally, compared to healthy individuals, AD patients exhibit elevated levels of pro-inflammatory cytokines (IL-1β, TNF-α, IL-6, etc.) and free radicals in the brain. These factors can further enhance Tau hyperphosphorylation and Aβ plaque deposition [11] because this chronic neuroinflammation leads to the misfolding of the Tau protein [16].

TNF-α (tumor necrosis factor-α), IL-1β (Interleukin-1β), and IL-6 (Interleukin-6) are the major pro-inflammatory cytokines in AD. According to reports [17], AD patients exhibit high levels of TNF-α in the brain, which promotes the expression of β- and γ-secretases. β-secretase cleaves APP to generate Aβ protein [18,19]. Additionally, mice lacking TNF-α in hybrid models with AD transgenic mice show reduced Aβ aggregation and diminished glial cell activity, leading to cognitive function restoration [20]. Therefore, patients with mild cognitive impairment accompanied by high levels of the pro-inflammatory cytokine TNF-α and low levels of the anti-inflammatory cytokine TGF-β in their cerebrospinal fluid (CSF) are considered to have a high tendency for AD development [21]. IL-1β is released in the early stages of Aβ deposition in the development of AD [22]. A significant presence of IL-1β can be observed in the brain tissue of AD patients. IL-1β is crucial for cellular defense and tissue repair in almost all tissues and is associated with pain, inflammation, and autoimmunity. IL-1β also participates in neuroprotection, tissue remodeling and repair, and regulation of the synthesis of APP and the process of amyloidosis [19]. Furthermore, elevated levels of IL-1β in AD patients promote the activation of mitogen-activated protein kinase (MAPK) signaling, ultimately leading to excessive phosphorylation of the Tau protein [23,24]. IL-6 is crucial for maintaining homeostasis in brain tissue, and its overexpression leads to chronic neuroinflammation [25]. Overexpression of IL-6 has been observed in brain tissue in AD mouse models (TgCRND8 and Tg2576) [26]. Similar to IL-1β, IL-6 can upregulate the mRNA expression of APP [27]. Additionally, IL-6 can induce excessive phosphorylation of the Tau protein by increasing the activator p53 of cyclin-dependent kinase 5 (CDK5), leading to neurofibrillary tangles [28]. COX-2 (cyclooxygenase-2) is an important enzyme involved in catalyzing the conversion of arachidonic acid to prostaglandins and is expressed during inflammatory processes. In a study on transgenic AD mouse models expressing human COX-2, the overexpression of COX-2 in neurons leads to the formation of Aβ plaques and the generation of free radicals, resulting in neuronal cell death and exacerbating cognitive deficits [29].

Reactive oxygen species (ROS), also known as oxygen free radicals (OFR), are generated during the normal cellular metabolic processes, such as cell oxidation, cellular regulation, and signal transduction [30]. At low to moderate concentrations, ROSs can play beneficial roles in regulating cellular processes, such as hormone regulation and intracellular secondary signal transduction [31,32]. However, at higher concentrations, they may cause harm to cellular lipids, protein expression, and DNA, ultimately leading to cell death [33,34,35]. Free radicals are highly reactive, inorganic, and unstable molecules or atoms that have lost an electron, leaving their outer valence shell incomplete [36]. Therefore, these incomplete oxygen-carrying intermediates, namely oxygen free radicals, can scavenge electrons from other molecules, and once paired, continue to generate more free radicals [32,37], thereby interacting with other molecules and leading to more complex cellular toxicity mechanisms [38]. Among them, the most destructive free radical in many tissues is the superoxide anion (O_2_^−^) [39], which is mainly produced in the respiratory chain of mitochondria [40] and is the primary source of toxicity caused by all free radicals [41,42]. O_2_^−^ is produced by the stepwise reduction of oxygen molecules. Thus, it can easily generate hydroxyl ion (OH^−^) radicals or hydroxyl free radicals (HFR), which can penetrate cell membranes and cause severe molecular damage, namely lipid peroxidation [43]. Existing studies have confirmed that, in the brains of AD patients, the excessive production of free radicals due to the imbalance of Aβ and Tau proteins leads to oxidative stress, exacerbating Aβ toxicity to neurons and causing neuronal glycosylation [44]. For example, high concentrations of free radicals can stimulate c-Jun N-terminal kinase and p38 mitogen-activated protein kinase, further promoting Aβ deposition [45]. Superoxide dismutase (SOD) is a metal enzyme and one of the major enzyme components responsible for scavenging superoxide free radicals, serving as the first line of defense in antioxidant defense mechanisms [46]. SOD can rapidly eliminate O_2_^−^, as it can reduce the oxidative second-order reaction to an oxidative first-order reaction [47]. However, if the concentration of free radicals is too high, O_2_^−^ can combine with NO to form peroxynitrite, or undergo the Fenton reaction to form OH^−^ radicals [48], with the latter being a stronger oxidant than O_2_^−^, causing greater damage to cells. Studies have observed that SOD knockout mice accelerate Aβ plaque deposition [49], increase Tau phosphorylation [50], and worsen behavioral deficits [51], all of which indicate the critical role of free radicals and SOD in human aging and AD.

GSK-3β (Glycogen synthase kinase-3β) is considered to be the most important protein kinase regulating Tau phosphorylation [52], GSK-3β has been shown to be highly associated with Aβ and Tau in the pathogenesis of AD [53], In Tet/GSK-3β mice with AD pathological features, restoration of normal GSK-3β activity after transgene shutdown resulted in normalized Tau phosphorylation levels, reduced neuronal death and reactive glial cell response, and improved cognitive deficits [54]. GSK-3β has been demonstrated to be a regulator of cholinergic function, as its activation affects cholinergic axonal transport in neurons [55].

In addition to the deposition of Aβ fibrils, NFTs, inflammatory reactions, and oxidative stress responses, the features of AD also include the loss of cholinergic neurons, the neurotransmitter acetylcholine (ACh), and its synthesizing enzymes, choline acetyltransferase, and acetylcholinesterase (AChE)-degrading enzyme [56,57,58,59,60].

AChE is a serine hydrolase widely present in various animal tissues and serum. At the synaptic cleft, AChE maintains the normal transmission of nerve impulses by catalyzing the hydrolysis of ACh, playing a crucial role in biological neural conduction. Acetylcholine has been implicated in the brain’s cognitive function and memory processing, with evidence suggesting abnormal activity of AChE and lower acetylcholine levels in AD patients compared to healthy individuals [61,62,63]. Similar to AChE, butyrylcholinesterase (BChE) is found in Aβ plaques and neurofibrillary tangles, promoting the proliferation of neurons and glial cells and regulating AChE expression. In contrast to AChE, enhanced activity of BChE has been observed in AD [56]. Aβ plaques and neurofibrillary tangles in AD have been shown to be associated with cholinesterase activity, especially BChE [64,65,66]. Studies have also found BChE involvement in the transition of Aβ amyloid deposition from benign to malignant states [67] because the reducing Aβ deposition has been observed in AD mouse models with BChE gene knockout [68,69,70].

Electroencephalogram (EEG) is used to measure the electrical signal activity of neuronal groups in the cerebral cortex, and specific electrical signal frequencies reflect changes in human emotions, such as excitement, anxiety, and calmness [71]. EEG signals are typically categorized into δ waves (0–4 Hz) associated with deep sleep and subconsciousness, θ waves (4–8 Hz) related to memory and drowsiness, α waves (8–13 Hz) associated with relaxation and tranquility, and β waves (13–30 Hz) related to concentration and cognition. Modern neurology research has found that, in early-stage Alzheimer’s disease (AD) patients, the power of α waves in the brain decreases, while in late-stage patients, both β and α waves weaken, leading to anxiety and cognitive impairment. However, the acetylcholinesterase inhibitor Rivastigmine can significantly increase the spectral power of α waves in AD patients [72], Clinically, AD patients receiving antipsychotic drug treatment have been found to develop drug resistance, with severe side effects occurring in the late stages of treatment, especially anxiety, agitation, or depression [73].

In recent years, the efficacy of inhaling essential oils as aromatherapy in effectively regulating human emotions has garnered increasing attention. The essential oils utilized in aromatherapy contain a large number of terpenoid molecules, which primarily exert pharmacological effects through two pathways: the bloodstream pathway and the neural pathway. In the bloodstream pathway, a portion of the small molecules of essential oils rapidly enter the capillaries through the alveoli, penetrate physiological barriers (such as mucous membranes, skin, and the blood–brain barrier), and interact with the central nervous system, exerting neuropharmacological effects [73]. In the neural pathway, another portion of the small molecules of essential oils binds to receptors on the dendrites of the olfactory sensory neurons (OSNs) in the olfactory epithelium, generating action potential signals transmitted via the olfactory nerve axis to the olfactory bulb. Subsequently, the signals are transmitted to mitral cells and tufted cells contacting the olfactory bulb and are relayed to the pyramidal neurons in the olfactory cortex, where the signals further stimulate the frontal lobe of the brain, ultimately reaching the limbic system. The limbic system can trigger emotional responses via the amygdala, stimulating the autonomic nervous system in the hypothalamus, thereby affecting the functions of various organs and so forth [74].

This study investigated the effects of DPEO on the expression levels of AD-related proteins (T-Tau, P-Tau, GSK-3β, Aβ_1–42_, COX-2, IL-1β, TNF-α, and IL-6) in neuronal cells using an in vitro AD cell model (Aβ_1–42_-induced damage in SH-SY5Y cells). Additionally, we explored the impact of DPEO on the activities of cholinergic-associated proteins (AchE and BchE) and oxidative reaction-related molecules (SOD activity and OFR and HFR scavenging ratio). Furthermore, human EEG experiments were conducted to investigate changes in four different brainwave bands in 25 males and 25 females after inhaling DPEO. Finally, through network pharmacology, the study delved into the mechanisms underlying the protective effects of DPEO on neuronal cells and its potential therapeutic value, providing strategies for alleviating the pathological processes of AD and other diseases.

## 2. Materials and Methods

### 2.1. In Vitro and In Vivo Materials

*Dalbergia pinnata* was purchased from Zhejiang Yifeng Cosmetics Co., Ltd. (Shaoxing, China). The essential oil extracted from *Dalbergia pinnata* was obtained using the supercritical carbon dioxide extraction method and stored at 4 °C until utilized. The supercritical fluid extractor (HA220-50-06, Nantong Huaan Supercritical Extraction Co., Ltd., Nantong, China) was employed for the extraction of the essential oil from *Dalbergia pinnata*. Under the conditions of pressure (35 MPa), temperature (75 °C), a carbon dioxide flow rate of 10 mL/min, and an extraction time of 60 min, the essential oil was successfully extracted. The samples were maintained in the Fragrance and Flavor Laboratory of Shanghai Institute of Technology.

**Figure 1:**
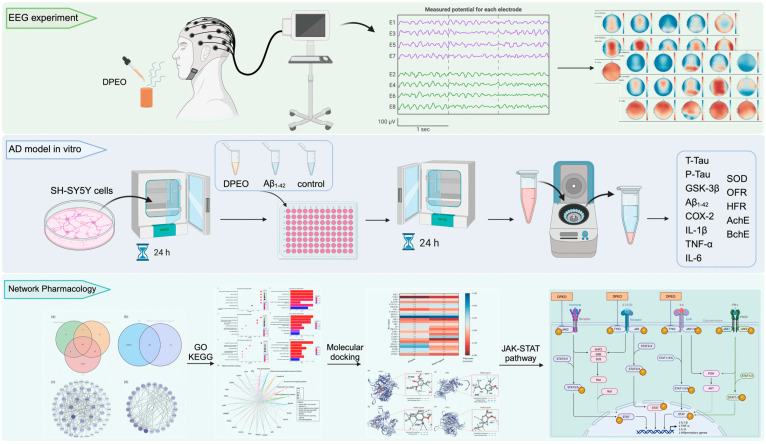
Main process of in vivo, in vitro, and network pharmacology.

Cell experimental materials and reagents were procured from Bioseth Biotechnology (Zhenjiang) Co., Ltd. (Zhenjiang, China), including SH-SY5Y cells, 0.25% Trypsin-EDTA, phosphate buffer saline (PBS), Dulbecco’s modified eagle medium (DMEM), fetal bovine serum (FBS), penicillin–streptomycin solution, DMSO, human Aβ_1–42_, Cell Counting Kit-8 (CCK-8), BchE activity assay kit, AchE activity assay kit, Oxygen Free Radical (OFR) scavenging capacity assay kit, Hydroxyl Free Radical (HFR) Scavenging Capacity assay kit, SOD activity assay kit, human IL-1β ELISA assay kit, human TNF-α ELISA assay kit, human IL-6 ELISA assay kit, human COX-2 ELISA assay kit, human GSK-3β ELISA assay kit, human P-Tau ELISA assay kit, human T-Tau ELISA assay kit, and human Aβ_1–42_ ELISA assay kit, among others. The main process of experiments are showed in Figure 1.

### 2.2. EEG Experiment Prepare

For this EEG experiment, a laboratory with a temperature of 25 °C, humidity of 70%, and noise level below 40 decibels, and equipped with a fresh air system, was selected. Concurrently, we recruited 25 healthy male and 25 healthy female volunteers, with an average age range of 20–30 years. Each participant was briefed on the experimental procedures and objectives and was required to abstain from consuming alcohol or caffeine-containing foods for 24 h before the start of the experiment, as well as refrain from taking psychotropic drugs within the past week.

This study adhered to the guiding principles of the Helsinki Declaration and the Tokyo Declaration and was approved by the Ethics Committee of Shanghai Jiao Tong University, with approval number #B2021153I, and obtained informed consent from all participants.

### 2.3. EEG Experiment Process

Prior to commencing the experiment, clean volunteers were fitted with an electrode cap containing 32 electrode channels adjusted to achieve a resistance below 5 Ω. EEG signals from four brain regions (the frontal, parietal, occipital, and temporal lobes) were recorded for 2 min each in both pre-stimulus and post-stimulus conditions using an EEG recording system (eggoTM mylab, ANT Neuro, Hengelo, The Netherlands) equipped with a 32-electrode configuration. The EEG signals were segmented into δ, θ, α, and β waves using fast Fourier transform (FFT) and visualized for analysis.

### 2.4. AD Cell Model Establishment and Cell Viability

In the initial experiments, SH-SY5Y cells were cultured in DMEM supplemented with 10% FBS and 1% penicillin-streptomycin at 37 °C in a 5% CO_2_ atmosphere for 2 days. The growth medium was refreshed every 12 h to maintain cells in the logarithmic growth phase. Cells in the logarithmic growth phase at a concentration of 1 × 10^5^/mL were seeded into 96-well cell-culture plates. The cells were divided into the following treatment groups: (1) introduction of Aβ_1–42_ at concentrations of 5, 10, 15, or 20 μM, followed by a 24 h incubation period at 37 °C in a 5% CO_2_ environment to establish the AD cell model group [75]; (2) treatment with DPEO at concentrations of 0.02%, 0.03%, 0.04%, 0.05%, or 0.06% (*v*/*v*) for 24 h under the same conditions to create the DPEO-treated cell group; (3) control group incubated for 24 h, model group (Aβ_1–42_ 10 μM) incubated for 24 h, and treated group with 10 μM Aβ_1–42_ and DPEO at 0.04% (*v*/*v*) added to the cells for 24 h incubation. Following the designated incubation periods, 10 μL of CCK-8 (10 nmol/L) were added to each well and incubated for 2 h at 37 °C. Cell viability was then determined by measuring the absorbance of the cells at a wavelength of 450 nm.

### 2.5. Morphological Observation

The SH-SY5Y cells in the logarithmic growth phase were seeded into 96-well plates and incubated at 37 °C in a 5% CO_2_ environment for 24 h. The cells were allocated into four groups: (a) control; (b) 0.04% DPEO (*v*/*v*); (c) AD model (Aβ_1–42_ 10 μM); and (d) treated group (Aβ_1–42_ 10 μM + 0.04% DPEO (*v*/*v*)). Following the addition of the agents, the cells were incubated for 24 h and, subsequently, observed and photographed under an inverted microscope.

### 2.6. Assay of AD Cells Model

Gently wash the cells to be tested with pre-chilled PBS and dissociate the cells using 0.25% Trypsin-EDTA. Centrifuge the cells at 1000 rpm for 5 min to collect them, discard the supernatant, and wash the cells three times with PBS. Resuspend the cells and sonicate them to break the cells (200 W, sonication for 3 s with a 7 s interval, totaling 3 min). Centrifuge at 3000 rpm and 4 °C for 10 min, and immediately collect the supernatant for further analysis according to the instructions provided by the reagent kit manufacturer.

### 2.7. Network Pharmacology Database and Analysis Platform

Network pharmacology analysis was conducted using online databases and platforms (Table 1). Initially, the major components of DPEO were matched with the Swiss target-prediction online database to obtain corresponding targets. Additionally, Alzheimer’s Disease-related targets were obtained from the human gene database Genecards, which contains rich gene data. Subsequently, these targets were depicted in a Venn diagram, and redundant targets were removed to obtain the drug AD targets. Key targets were selected through the STRING database and the Cytoscape 3.10.2 software (San Diego, MA, USA) based on Java 17.0.5. Furthermore, the key targets were subjected to a GO/KEGG analysis using the analysis tool Hiplot based on the R language.

The PubMed database was utilized to obtain the molecular structure of the major components, while the Uniprot database and the AlphaFold protein-structure database were used to obtain the protein structures of the key targets. Finally, the molecular docking of the major components with the protein structures of the key targets was conducted using the cavity detection guided blind docking analysis platform to determine the final key targets and to illustrate the theoretical pharmacological pathways.

### 2.8. Statistical Analysis

In vitro experiments were conducted in triplicate, and GraphPad Prism 10.2 statistics software was utilized for all statistical analyses. The data were presented as the means ± SD of three or more independent experiments, and significant differences were assessed by one-way analysis of variance (ANOVA). In cases of heteroscedasticity, Brown–Forsythe and Welch ANOVA tests were applied. Post hoc multiple comparisons were performed using Tukey’s test and Dunnett’s *t*-test (*p* < 0.05 was considered significant). The *t*-test (Student’s *t*-test) was employed to analyze the results of the electroencephalogram signals, where an absolute T-value > 2 was considered to be indicative of significant differences.

## 3. Results

### 3.1. EEG for Female and Male after Inhaling DPEO

**Figure 2:**
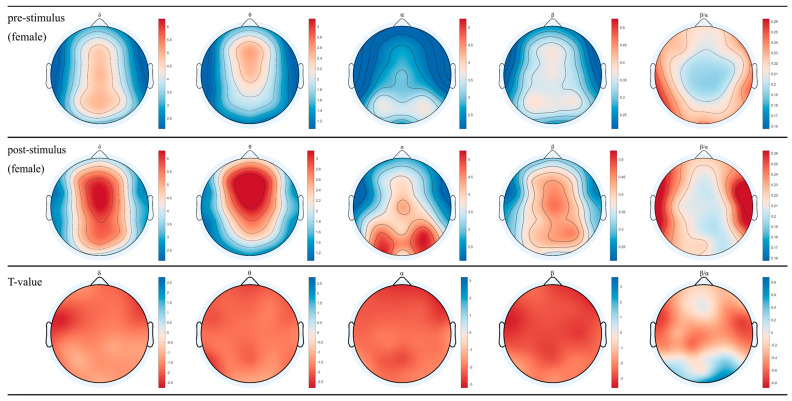
EEG topographical maps for females. The changes in power across the four brainwaves from left to right (δ, θ, α, β, and β/α) before and after inhaling DPEO.

**Figure 3:**
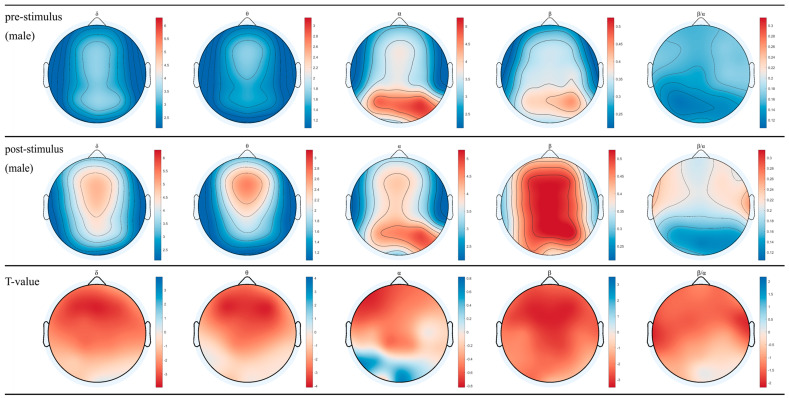
EEG topographical maps for males. The changes in power across the four brainwaves from left to right (δ, θ, α, β, and β/α) before and after inhaling DPEO.

The topographical maps of brainwaves (Figure 2 and Figure 3) illustrate the variations in δ, θ, α, and β waves across different brain regions in males and females. The ratio of β/α reflects the degree of arousal in the brain regions, with an increase in the β wave accompanied by a decrease in α waves, signifying heightened arousal. Following the inhalation of DPEO, the power of the four types of brain waves showed varying degrees of enhancement across different brain regions in both males and females. Significant enhancements in δ and θ waves were observed in the frontal and parietal lobes, indicating a state of deep relaxation. β waves exhibited significant enhancement across the entire brain, suggesting heightened focus and cognitive abilities, with a stronger enhancement in males. Enhanced β/α in the temporal lobe indicates increased arousal of auditory, memory, and emotional functions, which are notably more pronounced in males than females. Notably, α waves increased in the male frontal lobe but decreased in the occipital lobe, accompanied by an increase in β waves in the frontal and parietal lobes, indicating improved focus and cognitive function in males. In females, both α and β waves increased across the entire brain, with a significant enhancement of β waves in the frontal and parietal lobes, indicating relaxation alongside enhanced cognitive abilities in females.

### 3.2. SH-SY5Y Cell Viability

**Figure 4:**
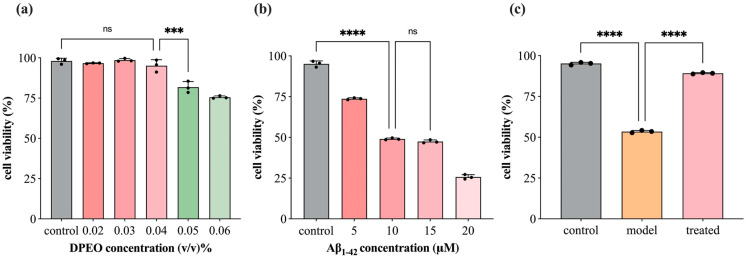
SH-SY5Y cell viability. (**a**) DPEO cytotoxicity towards SH-SY5Y cells, (**b**) Aβ_1–42_ cytotoxicity towards SH-SY5Y cells, (**c**) CCK-8 for control, model (10 μM Aβ_1–42_), and treated (10 μM Aβ_1–42_ and 0.04% DPEO) group. (*p*-value: ns (not significant), * < 0.05, *** < 0.001, **** < 0.0001).

Figure 4a illustrates that DPEO exhibits no significant cytotoxicity towards SH-SY5Y cells when the concentration is <0.04%. In Figure 4b, the treatment of SH-SY5Y cells with 10 μM Aβ_1–42_ resulted in an approximate LD_50_ in cell viability, with no significant difference observed compared to the 15 μM dosage group. Figure 4c demonstrates that treatment with 0.04% (*v*/*v*) DPEO followed by 10 μM Aβ_1–42_ led to a substantial increase in cell viability to approximately 90%. Conversely, under the conditions of 10 μM Aβ_1–42_ alone, the cell survival rate plummeted to 50%, showing significant deviation from the treated group. These initial findings prompted the selection of a DPEO concentration of 0.04% (*v*/*v*) for further investigation into the pharmacological mechanisms underlying the efficacy of this herbal medicine.

### 3.3. Morphological Changes of SH-SY5Y Cells

**Figure 5:**
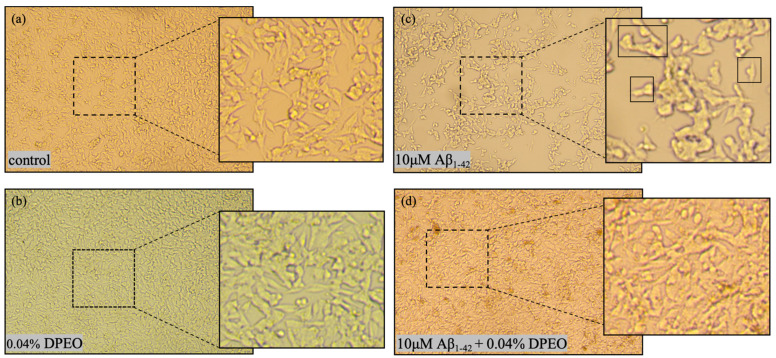
Morphological images of SH-SY5Y cells following different treatments. (**a**) Control represents the normal cellular morphology. (**b**) Cellular morphology after treatment with 0.04% DPEO. (**c**) Cellular morphology following treatment with 10 μM Aβ_1–42_ exhibiting rupture and shrinkage. (**d**) Cellular morphology after treatment with 10 μM Aβ_1–42_ + 0.04% DPEO (*v*/*v*) (treated group) displaying relatively good cellular morphology.

After exposure, the cellular morphology was examined using an inverted microscope. In both the control group and following exposure to 0.04% DPEO, the SH-SY5Y cells appeared large, bright, and well-defined and exhibited intact cell-membrane boundaries (Figure 5a,b). The majority of the control cells displayed a fusiform or olive shape. However, after 24 h of exposure to 10 μM Aβ_1–42_, the SH-SY5Y cells demonstrated characteristics such as cell body shrinkage, irregular shape, rough cell surface, a notable decrease in cell count, and significant amounts of cellular debris (Figure 5c). Furthermore, the Aβ_1–42_ treatment led to the retraction and potential disappearance of neurites. As depicted in Figure 5c, the neural pathway based on neurites was severely disrupted. The results presented in Figure 5d indicate that exposure of SH-SY5Y cells to 10 μM Aβ_1–42_ and 0.04% DPEO resulted in an increased cell count and a recovery of cellular morphology to levels similar to those of the control.

### 3.4. Protein Concentration Level of T-Tau, P-Tau, GSK-3β, Aβ_1–42_, COX-2, IL-1β, TNF-α, and IL-6

**Figure 6:**
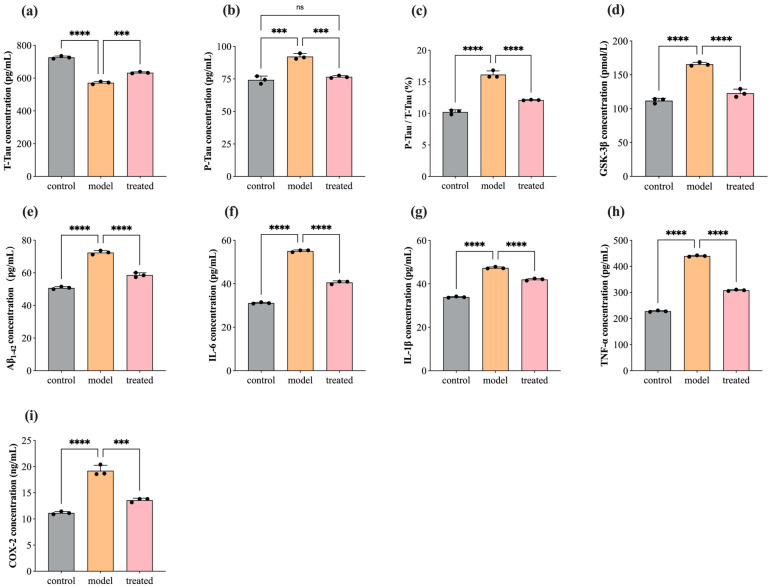
Concentration of T-Tau, P-Tau, GSK-3β, Aβ_1–42_, COX-2, IL-1β, TNF-α, and IL-6 in SH-SY5Y cells before and after treatment with 0.04% DPEO (*v*/*v*) at a concentration of 10 μM Aβ_1–42_. (**a**) The concentration of T-Tau, (**b**) the concentration of P-Tau, (**c**) P-Tau concentration/T-Tau concentration, (**d**) the concentration of GSK-3β, (**e**) the concentration of Aβ_1–42_, (**f**) the concentration of COX-2, (**g**) the concentration of IL-1β, (**h**) the concentration of TNF-α, and (**i**) the concentration of IL-6. (*p*-value: ns (not significant), *** < 0.001, **** < 0.0001).

In AD pathology, abnormal phosphorylation of Tau and deposition of Aβ_1–42_ around neurons are considered primary pathological features. Their presence disrupts normal neuronal cellular activities, activating the immune system to produce an abundance of inflammatory factors (COX-2, IL-1β, TNF-α, IL-6, etc.), leading to an inflammatory response that exacerbates the AD pathological process [76]. In this study, we investigated the concentration levels of T-Tau, P-Tau, GSK-3β, Aβ_1–42_, COX-2, IL-1β, TNF-α, and IL-6 in SH-SY5Y cells before and after treatment with 0.04% DPEO (*v*/*v*) at a concentration of 10 μM Aβ_1–42_. Figure 6 illustrates that, when SH-SY5Y cells are exposed to a concentration of 10 μM Aβ_1–42_, the expression levels of P-Tau, GSK-3β, Aβ_1–42,_ COX-2, IL-1β, TNF-α, and IL-6 in AD model cells are significantly elevated compared to the control group, while T-Tau expression levels are markedly reduced. Compared to the AD model, the treated group’s cells exhibit significantly reduced expression levels of P-Tau, GSK-3β, Aβ_1–42_, COX-2, IL-1β, TNF-α, and IL-6, with significantly increased T-Tau expression levels and no significant difference in P-Tau expression levels compared to the control group. Additionally, in Figure 6c, the ratio of P-Tau concentration to T-Tau concentration demonstrates the relative phosphorylation level of the Tau protein in neuronal cells, which is significantly lower in the treated group compared to AD model cells.

### 3.5. Antioxidant: SOD, OFR, and HFR

**Figure 7:**
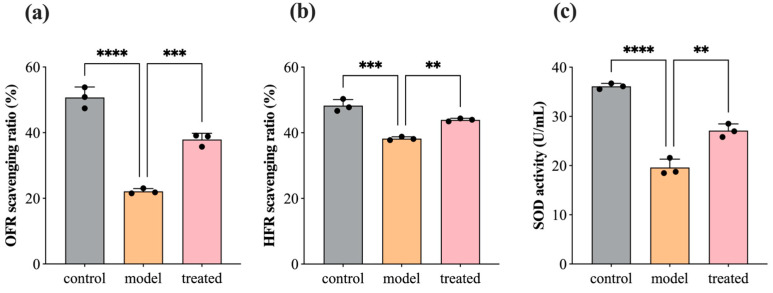
SH-SY5Y cells’ OFR scavenging ratio, HFR scavenging ratio, and SOD activity before and after treatment with 0.04% DPEO (*v*/*v*) at a concentration of 10 μM Aβ_1–42_. (**a**) OFR scavenging ratio, (**b**) HFR scavenging ratio, (**c**) SOD activity. (*p*-value: ** < 0.01, *** < 0.001, **** < 0.0001).

During the inflammatory response, cells generate a large amount of free radicals, mainly including reactive oxygen species (superoxide anion radicals, hydroxyl radicals, and nitric oxide radicals), which attack normal cell structures leading to neuronal damage. Superoxide dismutase (SOD), as a crucial enzyme involved in cellular oxidative reactions, serves as the first line of defense in the antioxidant defense mechanism. Figure 7 illustrates the scavenging ratio of OFR and HFR and the activity of SOD in SH-SY5Y cells before and after treatment with 0.04% DPEO (*v*/*v*) at a concentration of 10 μM Aβ_1–42_. In AD model cells, the OFR scavenging ratio, HFR scavenging ratio, and SOD activity show significant decreases. In comparison to the AD model, the treated group’s cells exhibit significant increases in the OFR scavenging ratio, HFR scavenging ratio, and SOD activity. Lower levels of free radicals and enhanced SOD activity are beneficial for maintaining the stability of normal cell structures, reducing inflammatory responses, and slowing down the progression of AD pathology.

### 3.6. DPEO Inhibits Activity of AchE and BchE

**Figure 8:**
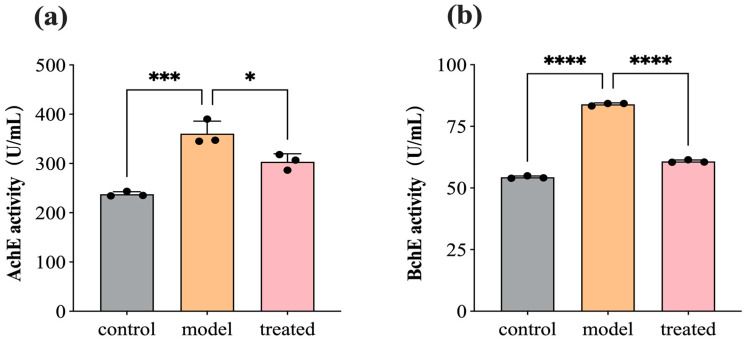
AchE and BchE activity in SH-SY5Y cells before and after treatment with 0.04% DPEO (*v*/*v*) at a concentration of 10 μM Aβ_1–42_. (**a**) AchE activity, (**b**) BchE activity. (*p*-value: * < 0.05, *** < 0.001, **** < 0.0001).

AchE and BchE serve as crucial hydrolytic enzymes in regulating neurotransmitters by catalyzing the hydrolysis of acetylcholine and butyrylcholine in the synaptic cleft to modulate neuronal impulses. Excessive activity of cholinesterases can diminish signal transmission between neurons, leading to cognitive impairments, such as memory loss and aphasia. Therefore, acetylcholinesterase inhibitors are among the primary drugs used in the treatment of Alzheimer’s disease (AD). Figure 8 depicts the activity of AchE and BchE in SH-SY5Y cells before and after treatment with 0.04% DPEO (*v*/*v*) at a concentration of 10 μM Aβ_1–42_. In AD model cells, AchE and BchE activity significantly increases. In comparison to the AD model, the treated group’s cells exhibit a significant decrease in AchE and BchE activity, indicating that DPEO markedly inhibits the activity of AchE and BchE.

### 3.7. Network Pharmacology Analysis

#### 3.7.1. Screen Targets of AD and Main Components of DPEO

**Figure 9:**
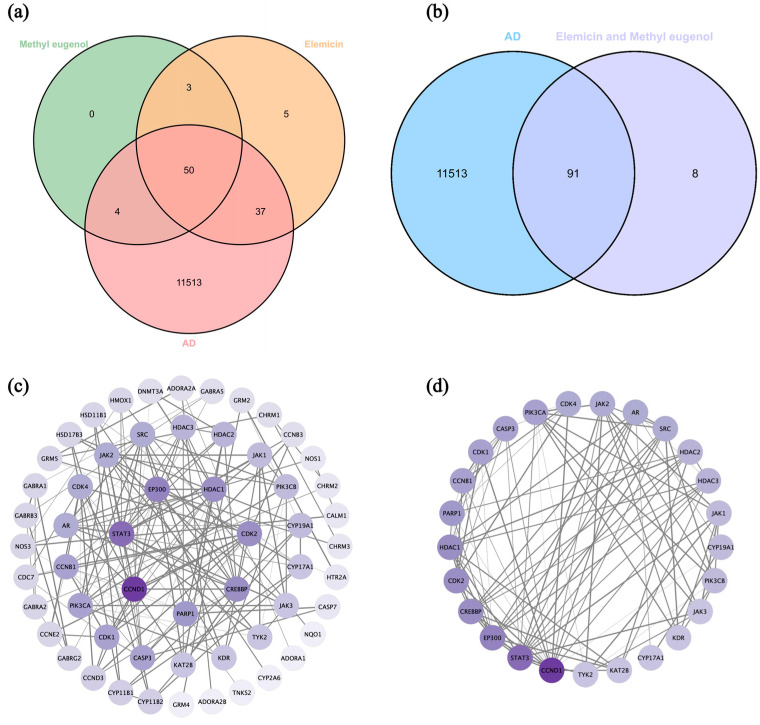
(**a**) Venn diagram of intersection targets of elemicin, methyl eugenol, and Alzheimer’s disease. (**b**) Venn diagram of intersection targets of elemicin and methyl eugenol for Alzheimer’s Disease. (**c**) Drug AD targets PPI network. (**d**) Key targets PPI network.

In our previous study [71], it was found that the major components in DPEO are elemicin (84.10%) and methyl eugenol (11.19%), together constituting 95.29% of the volatile components. Through the Swiss Target Prediction online database, 95 target proteins corresponding to elemicin and 57 target proteins corresponding to methyl eugenol were obtained. Additionally, 9344 AD-related targets (relevance score > 1.0) were retrieved from the Genecards database. Subsequently, a Venn diagram was constructed to identify the intersection of these targets (Figure 9a), and redundant targets were removed, resulting in 91 drug AD targets (Figure 9b). A protein–protein interaction (PPI) network of the drug AD targets was constructed using the STRING database with a high confidence score (>0.9) and further visualized using Cytoscape 3.10.2. The average degree of the drug AD targets in the PPI network was calculated to be 5.82, and 25 key targets (degree > 5.8) were selected (Table 2). Subsequently, a PPI network specifically depicting the key targets was generated (Figure 9d). In the PPI network, the thicknesses of the edges between targets represent the strength of their interactions.

#### 3.7.2. Key Targets GO/KEGG Analysis and Molecular Docking

**Figure 10:**
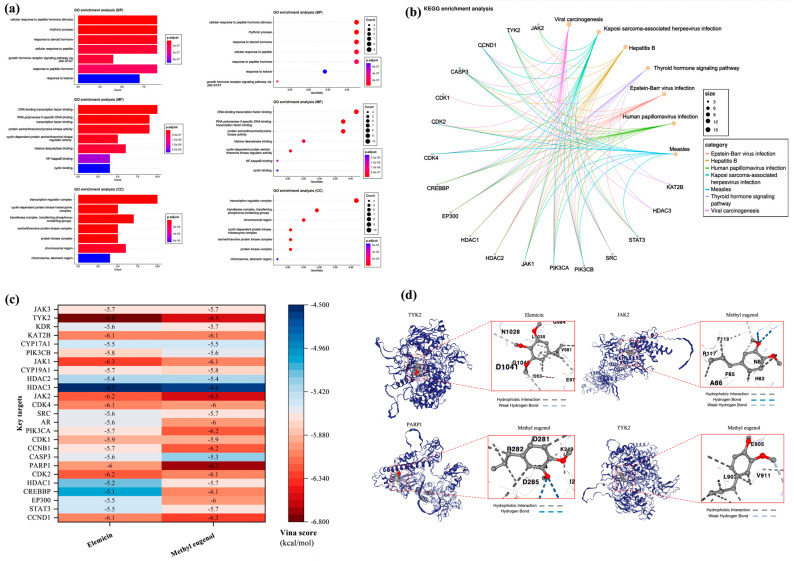
Key targets GO/KEGG enrichment analysis and molecular docking. (**a**) GO function enrichment analysis. (**b**) KEGG pathway enrichment analysis. (**c**) Heatmap of molecular docking. They are elemicin-TYK2 methyl eugenol-PARP1, methyl eugenol-JAK2, and methyl eugenol-TYK2. (**d**) Structure of molecular docking (Vina score is ≤−5.0 kcal/mol, the molecular binding is relatively stable, Vina score is ≤−7.0 kcal/mol, and the molecular binding is highly stable).

Hiplot was used to perform GO function and KEGG pathway enrichment analysis on key targets, yielding 1629 data entries. The data were classified based on biological process (BP), molecular function (MF), cellular component (CC), and KEGG pathways, and the top seven highly enriched entries for each category were selected for presentation (Figure 10a,b). In terms of biological process, the enriched entries mainly involve the cellular response to a peptide hormone stimulus, rhythmic process, response to steroid hormone, cellular response to peptide, response to peptide hormone, response to ketone, and growth-hormone receptor signaling pathway via JAK-STAT. For molecular function, the enriched entries primarily include DNA-binding transcription factor binding, RNA polymerase II-specific DNA-binding transcription factor binding, protein serine/threonine/tyrosine kinase activity, histone deacetylase binding, cyclin-dependent protein serine/threonine kinase regulator activity, NF-kappaB binding, and cyclin binding. In the context of the cellular component, the enriched entries are related to the transcription regulator complex, transferase complex (transferring phosphorus-containing groups), chromosomal region, cyclin-dependent protein kinase holoenzyme complex, serine/threonine protein kinase complex, protein kinase complex, and chromosome (telomeric region). Finally, the enriched KEGG pathways are primarily associated with Epstein–Barr virus infection, hepatitis B, human papillomavirus infection, Kaposi sarcoma-associated herpesvirus infection, measles, thyroid hormone signaling pathway, and viral carcinogenesis.

The molecular structures of elemicin and methyl eugenol were obtained from the PubMed database. Subsequently, the protein structures of 25 key targets were retrieved from the Uniprot database and the AlphaFold protein-structure database. These structures were then subjected to molecular docking with elemicin and methyl eugenol using the cavity-detection guided blind docking analysis platform (Figure 10c). Based on the Vina score, which describes the binding affinity of the molecules, four stronger molecular-protein complexes were identified: “Methyl eugenol-PARP1” (−6.7 kcal/mol), “Elemicin-TYK2” (−6.8 kcal/mol), “Methyl eugenol-JAK2” (−6.5 kcal/mol), and “Methyl eugenol-TYK2” (−6.5 kcal/mol). Subsequent analysis of the binding sites of these two complexes (Figure 10d) revealed that the “Elemicin-TYK2” complex docks through hydrophobic interaction and weak hydrogen bonding, while the “Methyl eugenol-PARP1” complex docks through hydrophobic interaction and hydrogen bonding. Additionally, the “Methyl eugenol-JAK2” complex docks through hydrophobic interaction, hydrogen bonding, and weak hydrogen bonding, and the “Methyl eugenol-TYK2” complex docks through hydrophobic interaction and weak hydrogen bonding.

#### 3.7.3. JAK-STAT Signaling Pathway

**Figure 11:**
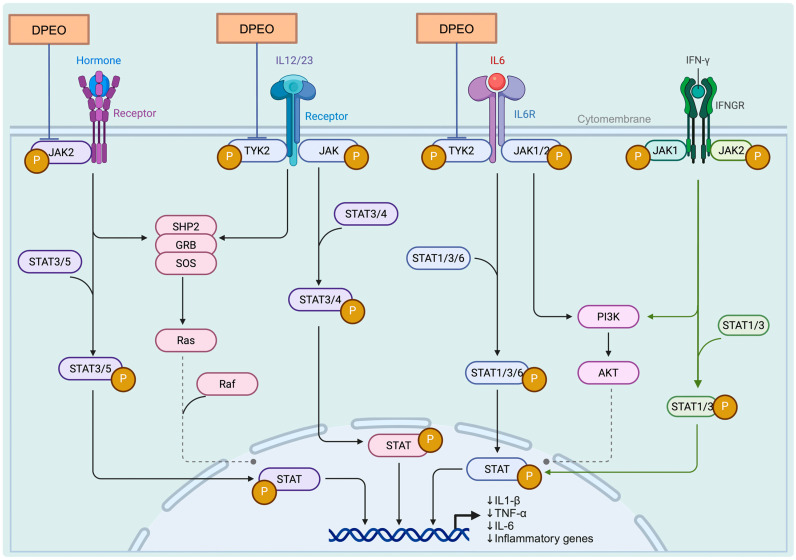
DPEO in JAK-STAT signaling pathway.

In the KEGG database, we identified the JAK-STAT signaling pathway as highly relevant to TYK2, JAK2, and PARP1, and a theoretical pharmacological pathway of DPEO was illustrated (Figure 11). The Janus kinase-signal transducers and activators of the transcription (JAK-STAT) pathway are a conserved signaling cascade utilized by various organisms for signal transduction in processes such as development and homeostasis. In mammals, this pathway serves as a direct route from the cell membrane to the nucleus, activated by cytokines, growth factors, and interferons, as well as other extracellular factors that regulate gene expression [77]. Upon cytokine binding to their specific receptors, members of the JAK family of tyrosine kinases activate STATs, leading to their dimerization, nuclear translocation, and subsequent modulation of target gene expression. This pathway plays a crucial role in cellular responses to environmental stimuli, governing cell growth, differentiation, proliferation, apoptosis, and inflammatory processes [78]. In addition to STAT activation, JAKs facilitate the recruitment of molecules such as MAP kinases and PI3 kinase, which process downstream signals through pathways like Ras-Raf-MAP kinase and PI3 kinase, ultimately activating additional transcription factors.

TYK2 (tyrosine-protein kinase 2) is a non-receptor protein kinase that belongs to the JAK family and plays a crucial role in various diseases, including psoriasis, inflammatory bowel disease, and systemic lupus erythematosus. TYK2 activates downstream proteins STAT1-5 by engaging in the signal transduction of immune factors such as IL-12, IL-23, and IL-10, thereby modulating immune responses. Inhibiting the activity of TYK2 can effectively disrupt the transmission of excessive immune signals and serve as a promising therapeutic approach for the treatment of these diseases [79]. PARP1 (Poly (ADP-Ribose) Polymerase 1), the most extensively studied isoform within the nuclear enzyme PARP family, exerts a pivotal influence on diverse cellular biological processes, including DNA repair and gene transcription. Additionally, PARP1 has been implicated in the pathogenesis of several carcinomas due to its overexpression in these malignancies [80]. JAK2 (Janus Kinase 2) participates in the signal transduction of cellular immune factors, activating the JAK-STAT pathway. This pathway is characteristic of certain malignant tumors, as well as inflammatory or immune diseases, including chronic bone-marrow proliferative disorders and bone-marrow fibrosis [81]. We particularly believe that this is the potential pharmacological mechanism through which Dalbergia pinnata alleviates.

## 4. Discussion

*Dalbergia pinnata*, a traditional Chinese aromatic medicinal plant, finds widespread application in wound healing and cardiovascular, and respiratory diseases. Its active constituents primarily consist of volatile oils, including elemicin and methyl eugenol, with relative contents of 84.95% and 11.19%, respectively [71]. However, research on its neurological effects remains limited.

Alzheimer’s Disease (AD) is a prevalent neurodegenerative cognitive disorder in populations worldwide, with projections by the WHO estimating the number of AD patients to reach 100 million by 2050. The pathogenesis of Alzheimer’s Disease is believed to be highly associated with the deposition of Aβ and the excessive phosphorylation of Tau protein leading to NFTs, which is exacerbated by the overexpression of inflammation [16]. Currently, there is no cure for AD, and available treatments primarily focus on acetylcholinesterase inhibitors and NMDA receptor antagonists, which aim to alleviate symptoms and improve memory and daily functioning [82]. Additionally, depression and anxiety are commonly observed in AD patients, with reports of resistance to antipsychotic drug therapy and the emergence of severe side effects, particularly anxiety, agitation, or depression during late-stage treatment [73]. Aromatherapy, as a natural therapy, utilizes aromatic molecules from plants that exert pharmacological effects upon inhalation, acting through both the blood and neural pathways, with emotional regulation being a prominent effect [73,74].

Through the analysis of EEG data from 50 human subjects, it was observed that the inhalation of DPEO resulted in significant enhancements of δ and θ waves in the frontal and parietal lobes for both male and female participants. Moreover, there was a significant increase in β waves across the entire brain, indicating a state of deep relaxation and enhanced brain focus and cognitive abilities. The increased β/α ratio in the temporal lobe suggests heightened levels of auditory, memory, and emotional function arousal. Notably, differences were observed in the patterns of α waves between the male and female participants. In males, there was an increase in α waves in the frontal lobe and a decrease in the occipital lobe, accompanied by enhanced β waves in the frontal and parietal lobes, signifying improved concentration and cognitive functions. Conversely, in females, both α and β waves were enhanced across the entire brain, with a significant increase in β waves in the frontal and parietal lobes, indicating enhanced cognitive abilities alongside relaxation.

In addition, experimental findings using DPEO treatment on Aβ_1–42_-damaged SH-SY5Y cells, a cellular model of AD, demonstrated that DPEO could elevate Tau protein levels and reduce the phosphorylation of Aβ_1–42_ and P-Tau proteins within neuron cells to maintain normal neuronal structures. Furthermore, DPEO was shown to downregulate the levels of inflammatory factors IL-1β, TNF-α, IL-6, and COX-2, thereby reducing neuronal inflammatory responses. Additionally, DPEO enhanced the activity of SOD enzymes in neuron cells and increased the scavenging ratio of OFR and HFR to sustain neuronal antioxidant capacity, inhibiting AchE and BchE activities to uphold normal neuronal signal transmission. Through network pharmacology analysis, it was revealed that DPEO may exert its pharmacological activity by interacting with the JAK-STAT signaling pathway. Particularly, four stronger binding complexes (elemicin-TYK2 methyl eugenol-PARP1, methyl eugenol-JAK2, and methyl eugenol-TYK2) were identified, suggesting significant pharmacological potential for their therapeutic effects. A review article [83] has reported that certain plant compounds can directly modulate the JAK-STAT signaling pathway in three ways, namely by (1) inhibiting the phosphorylation of JAK and/or STAT; (2) suppressing the activation and expression of JAK and/or STAT; (3) interfering with the movement and nuclear translocation of STAT, thus impacting the regulation of STAT target genes. Concurrently, we have also identified other potential pharmacological effects of DPEO, such as its anticancer, antiviral, and anti-inflammatory properties, and its ability to maintain normal cellular functions. As mentioned earlier in the article, Dalbergia pinnata has long been used in traditional medicine for the treatment of injuries and the prevention of respiratory diseases.

Despite the complex and diverse pathogenic mechanisms of Alzheimer’s disease, our present study provides compelling evidence supporting the neuropharmacological effects of Dalbergia pinnata, particularly in ameliorating the pathological features of Alzheimer’s disease.

## 5. Conclusions

In this study, EEG experiments revealed that inhalation of DPEO by human subjects can lead to the relaxation of the brain, attenuation of anxiety, and improvement of cognitive abilities. It was further observed through an in vitro AD cell model that DPEO significantly improves the pathological progression of AD by reducing AD pathological products, lowering inflammatory responses, enhancing neuronal antioxidant capacity, and maintaining neuronal signal transduction. Furthermore, a network pharmacology analysis suggested that DPEO may exert its pharmacological effects through the JAK-STAT signaling pathway.

## Figures and Tables

**Table 1:** Database and analysis platform for network pharmacology.

Category	Name	Website
Database	TCMSP	https://www.tcmsp-e.com/#/database (accessed on 4 December 2023)
	PubChem	https://pubchem.ncbi.nlm.nih.gov (accessed on 4 December 2023)
	Uniprot	https://www.uniprot.org (accessed on 10 December 2023)
	Swiss Target Prediction	http://www.swisstargetprediction.ch (accessed on 10 December 2023)
	GeneCards	https://www.genecards.org (accessed on 15 December 2023)
	STRING	https://string-db.org/ (accessed on 15 December 2023)
	KEGG	https://www.kegg.jp/ (accessed on 15 December 2023)
	AlphaFold	https://alphafold.ebi.ac.uk (accessed on 17 December 2023)
Analysis platform	CB-Dock2	https://cadd.labshare.cn/cb-dock2/index.php (accessed on 19 December 2023)
	Hiplot	https://hiplot.com.cn/home/index.html (accessed on 19 December 2023)

**Table 2:** Key targets.

NO.	Key Targets	Name	Uniprot ID	Degree
1	CCND1	Cyclin D1	P24385	19
2	STAT3	Signal Transducer And Activator Of Transcription 3	P40763	15
3	EP300	E1A Binding Protein P300	Q09472	13
4	CREBBP	CREB Binding Protein	Q92793	12
5	HDAC1	Histone Deacetylase 1	Q13547	12
6	CDK2	Cyclin Dependent Kinase 2	P24941	12
7	PARP1	Poly (ADP-Ribose) Polymerase 1	P09874	11
8	CASP3	Caspase 3	P42574	10
9	CCNB1	Cyclin B1	P14635	10
10	CDK1	Cyclin Dependent Kinase 1	P06493	10
11	PIK3CA	Phosphatidylinositol-4, 5-Bisphosphate 3-Kinase Catalytic Subunit Alpha	P42336	10
12	AR	Androgen Receptor	P10275	9
13	SRC	SRC Proto-Oncogene, Non-Receptor Tyrosine Kinase	P12931	9
14	CDK4	Cyclin Dependent Kinase 4	P11802	9
15	JAK2	Janus Kinase 2	O60674	9
16	HDAC3	Histone Deacetylase 3	O15379	8
17	HDAC2	Histone Deacetylase 2	Q92769	8
18	CYP19A1	Cytochrome P450 Family 19 Subfamily A Member 1	P11511	7
19	JAK1	Janus Kinase 1	P23458	7
20	PIK3CB	Phosphatidylinositol-4, 5-Bisphosphate 3-Kinase Catalytic Subunit Beta	P42338	7
21	CYP17A1	Cytochrome P450 Family 17 Subfamily A Member 1	P05093	6
22	KAT2B	Lysine Acetyltransferase 2B	Q92831	6
23	KDR	Kinase Insert Domain Receptor	P35968	6
24	TYK2	Tyrosine Kinase 2	P29597	6
25	JAK3	Janus Kinase 3	P52333	6

## Data Availability

All data and materials are available upon request from the corresponding author.
